# Efficient Colonization and Therapy of Human Hepatocellular Carcinoma (HCC) Using the Oncolytic Vaccinia Virus Strain GLV-1h68

**DOI:** 10.1371/journal.pone.0022069

**Published:** 2011-07-11

**Authors:** Ivaylo Gentschev, Meike Müller, Marion Adelfinger, Stephanie Weibel, Friedrich Grummt, Martina Zimmermann, Michael Bitzer, Martin Heisig, Qian Zhang, Yong A. Yu, Nanhai G. Chen, Jochen Stritzker, Ulrich M. Lauer, Aladar A. Szalay

**Affiliations:** 1 Genelux Corporation, San Diego Science Center, San Diego, California, United States of America; 2 Department of Biochemistry, University of Wuerzburg, Wuerzburg, Germany; 3 Rudolf Virchow Center for Experimental Biomedicine, University of Wuerzburg, Wuerzburg, Germany; 4 Institute for Molecular Infection Biology, University of Wuerzburg, Wuerzburg, Germany; 5 Department of Gastroenterology and Hepatology, Medical University Hospital, Tuebingen, Germany; 6 Department of Internal Medicine, School of Medicine,Yale University, New Haven, Connecticut, United States of America; 7 Department of Radiation Oncology, Rebecca and John Moores Comprehensive Cancer Center, University of California San Diego, La Jolla, California, United States of America; Florida International University, United States of America

## Abstract

Virotherapy using oncolytic vaccinia virus strains is one of the most promising new strategies for cancer therapy. In this study, we analyzed for the first time the therapeutic efficacy of the oncolytic vaccinia virus GLV-1h68 in two human hepatocellular carcinoma cell lines HuH7 and PLC/PRF/5 (PLC) in cell culture and in tumor xenograft models. By viral proliferation assays and cell survival tests, we demonstrated that GLV-1h68 efficiently colonized, replicated in, and did lyse these cancer cells in culture. Experiments with HuH7 and PLC xenografts have revealed that a single intravenous injection (i.v.) of mice with GLV-1h68 resulted in a significant reduction of primary tumor sizes compared to uninjected controls. In addition, replication of GLV-1h68 in tumor cells led to strong inflammatory and oncolytic effects resulting in intense infiltration of MHC class II-positive cells like neutrophils, macrophages, B cells and dendritic cells and in up-regulation of 13 pro-inflammatory cytokines. Furthermore, GLV-1h68 infection of PLC tumors inhibited the formation of hemorrhagic structures which occur naturally in PLC tumors. Interestingly, we found a strongly reduced vascular density in infected PLC tumors only, but not in the non-hemorrhagic HuH7 tumor model. These data demonstrate that the GLV-1h68 vaccinia virus may have an enormous potential for treatment of human hepatocellular carcinoma in man.

## Introduction

Hepatocellular carcinoma (HCC) is one of the most common malignancies worldwide [1], [2]. Despite progress in the diagnosis and treatment of HCC, overall patient treatment outcome has not substantially improved in the past. Therefore, the development of new therapies for HCC is a high priority. One of the most promising novel cancer therapies is oncolytic virotherapy. This method is based on the capacity of oncolytic viruses (OVs) to preferentially infect and lyse cancer cells. At the moment, several OV platforms (vaccinia virus, herpes simplex virus and reovirus) are in or entering Phase III clinical trials.

Here, we have investigated the therapeutic potential of the oncolytic vaccinia virus GLV-1h68 against HCC in preclinical studies. GLV-1h68 was derived from vaccinia virus Lister strain (LIVP) that contains an inactive thymidine kinase (tk) gene and shows inherently more tumor-selective replication than vaccinia virus WR strain [3], [4]. The virus strain GLV-1h68 was engineered by inserting 3 expression cassettes encoding a) *Renilla* luciferase-green fluorescent protein (Ruc-GFP) fusion protein into the F14.5L locus, b) ß-galactosidase into the thymidine kinase (tk) locus, and c) ß-glucuronidase into the hemagglutinin locus in the genome of the LIVP strain. The insertion resulted in highly attenuated virus strain compared to the wild-type parental strain [3].

We and others have already demonstrated tumor selectivity and efficacy of GLV-1h68 in many different tumor xenograft models, including human breast cancer [3], anaplastic thyroid carcinoma [5], [6], malignant pleural mesothelioma [7], pancreatic tumor [8], prostate carcinoma [9], squamous cell carcinoma [10], and canine breast cancer [11], [12]. In addition, Kelly et al. reported that GLV-1h68 virus could be used as a tool for detection of melanoma lymph node metastases in an immunocompetent animal model [13]. More recently, a GLV-1h68 derivative (GLV-1h99) that expresses the human norepinephrine transporter was shown to be useful for both therapy and deep-tissue imaging of tumors [14], [15].

Here, we describe that GLV-1h68 was able to infect, replicate in, and lyse human hepatocellular carcinoma cell lines HuH7 and PLC/PRF/5.

We also found that a single intravenous injection of GLV-1h68 into mice with subcutaneously grown hepatocellular carcinoma xenografts dramatically reduced tumor growth. Lastly, the oncolytic and immunological effects of GLV-1h68 in HCC tumors were analyzed by fluorescence imaging, immunohistochemistry, flow cytometry (FACS) and immune-related protein antigen profiling.

## Materials and Methods

### Ethics statement

All animal experiments were approved by the government of Unterfranken, Germany, and conducted according to the German animal protection guidelines (permit number: 55.2–2531.01-17/08).

### Cell lines

African green monkey kidney fibroblasts (CV-1, American Type Culture Collection, ATCC-No. CCL-70) and two human hepatocellular carcinoma cell lines HuH7 (ATCC CCL-185) and PLC/PRF/5 (PLC; ATCC CRL 8024) were maintained in Dulbecco's modified Eagle's medium (DMEM) supplemented with antibiotics (100 units/ml penicillin G, 100 units/ml streptomycin) and 10% fetal bovine serum (FBS; Invitrogen GmbH, Karlsruhe, Germany) at 37°C under 5% CO_2_.

### Virus strain

GLV-1h68 is a genetically stable oncolytic virus strain designed to locate, enter, colonize and destroy cancer cells without harming healthy tissues or organs [3].

### Cell viability assay with GLV-1h68

HuH7 and PLC cells were seeded onto 24-well plates (Nunc, Wiesbaden, Germany). After 24 h in culture, cells were infected with GLV-1h68 using multiplicities of infection (MOI) of 0.1 and 1. Cells were incubated at 37°C for 1 h, then the infection medium was removed and the cells were incubated in fresh growth medium. The amount of viable cells after infection with GLV-1h68 was measured using 3-(4,5-dimethylthiazol-2-yl)-2,5-diphenyltetrazolium bromide (MTT) (Sigma, Taufkirchen, Germany). At 24, 48, 72, or 96 h after infection of cells, medium was replaced by 0.5 ml MTT solution at a concentration of 2.5 mg/ml MTT dissolved in RPMI 1640 without phenol red and incubated at 37C° for 2 h in a 5% CO_2_ atmosphere. After removal of the MTT solution, the color reaction was stopped by adding 1 N HCl diluted in isopropanol. The optical density was then measured at a wavelength of 570 nm. Uninfected cells were used as reference and were considered as 100% viable. The amount of viable cells after infection with GLV-1h68 was measured in triplicates.

### Viral replication

HuH7 and PLC cells grown in 24-well plates were infected with GLV-1h68 at an MOI of 0.1. After incubation at 37°C for 1 h with gentle agitation every 20 min, the infection medium was removed and replaced by fresh growth medium. Supernatants were collected from virally treated cells at 1, 6, 12, 24, 48, 72 or 96 h post-infection. Serial dilutions of supernatants were titrated by standard plaque assays on CV-1 cells. All samples were measured in triplicates.

### Fluorescence imaging

The GFP signals of virus-infected cells were analyzed with a fluorescence microscope (Leica DM IRB; Wetzlar, Germany). Images were captured with an electronic camera and were processed using META-MORPH (Universal Imaging; Downingtown, PA, USA) and Photoshop 7.0 (Adobe Systems, Mountain View, CA, USA).

### GLV-1h68-mediated therapy of HuH7 and PLC xenografts

Tumors were generated by implanting hepatoma cells HuH7 or PLC (5×10^6^ cells in 100 µl of PBS) subcutaneously on the right flank above the hind leg of 6- to 8-week-old male or female nude mice (NCI/Hsd/Athymic Nude-*Foxn1* ^nu^, Harlan Winkelmann GmbH, Borchen, Germany). Tumor growth was recorded twice a week using a digital caliper. Tumor volume was calculated as [(length x width^2^)/2]. On day 10 post implantation, a single dose of the GLV-1h68 virus (5×10^6^ plaque forming units [pfu] in 100 µl PBS) was injected into the tail vein (i.v.) of HuH7 or PLC tumor-bearing mice. The animals of the control groups were injected i.v. with PBS only.

The statistical significance of the data was calculated by two-way analysis of variance (ANOVA) with Bonferroni comparison post-test (GraphPad Prism software, San Diego, USA). The post-test was only performed when ANOVA revealed significance. Results are displayed as means ± s.d. *P* values of <0.05 were considered significant.

### Histological analysis of tumors

For histological studies, tumors were excised and snap-frozen in liquid N_2_, followed by fixation in 4% paraformaldehyde/PBS at pH 7.4 for 16 h at 4°C. Tissue sectioning was performed as described by Weibel et al. [16]. GLV-1h68 was labeled using polyclonal rabbit anti-vaccinia virus (anti-VACV) antibody (Abcam, Cambridge, UK), which was stained using Cy3-conjugated donkey anti-rabbit secondary antibodies obtained from Jackson ImmunoResearch (West Grove, PA, USA). Phalloidin-TRITC (Sigma Aldrich, Taufkirchen, Germany) was used to label actin and Hoechst 33342 to label nuclei in tissue sections.

Endothelial cells were labeled with monoclonal rat anti-mouse CD31 antibody (BD Pharmingen, San Diego, CA) or hamster anti-mouse CD31 antibody (Chemicon, International, Temecula, CA). Immune cells were labeled using rat anti-mouse MHCII antibody detecting a polymorphic determinant present on B cells, monocytes, macrophages and dendritic cells (eBioscience, San Diego, CA). The Cy3- or Cy5-conjugated secondary antibodies (donkey) were obtained from Jackson ImmunoResearch (West Grove, PA).

The fluorescence-labeled preparations were examined using the MZ16 FA Stereo-Fluorescence microscope (Leica) equipped with the digital DC500 CCD camera and the Leica IM1000 4.0 software (1300×1030 pixel RGB-color images) as well as the Leica TCS SP2 AOBS confocal laser microscope equipped with an argon, helium-neon and UV laser and the LCS 2.16 software (1024×1024 pixel RGB-color images). Digital images were processed with Photoshop 7.0 (Adobe Systems, Mountain View, CA, USA) and merged to yield overlay images.

### Measurements of microvessel density

The vascular density was determined in microscopic images (x10 objective, x10 ocular, tissue region 1445 µm by 1445 µm) of CD31-labeled tumor sections captured with identical settings using the Leica TCS SP2 AOBS confocal laser microscope. All images were decorated with eight horizontal lines at identical positions using Photoshop 7.0 and all vessels which intersected these lines were counted to yield the vascular density. The vascular density was calculated for six images per group and presented as mean values ± standard deviations (s.d.).

### Preparation of tumor lysates for a mouse immune-related protein profiling

For preparation of tumor lysates, at 10 days after virus treatment, three mice from each group were sacrificed. Tumors were removed, resuspended in 9 volumes (W/V) lysis buffer [50 mM Tris-HCl (pH 7.4), 2 mM EDTA (pH 7.4), 2 mM PMSF and Complete Mini protease inhibitors (Roche, Mannheim, Germany)] and lysed using FastPrep FP120 Cell Disruptor (BIO 101, Qbiogene, Germany) at a speed of 6 for 20 s (three times). Samples were then centrifuged at 20,000 g at 4°C for 5 min and the supernatants were analyzed for mouse immune-related protein antigen profiling by Multi-Analyte Profiles (mouse MAPs; Rules Based Medicine, Austin, USA) using antibody linked beads. Results were normalized based on total protein concentration.

### Flow cytometric (FACS) analysis

For flow cytometric analysis, three or four mice from each group were sacrificed by CO_2_ inhalation and the tumors were removed. The tumor tissues were minced and incubated individually in 10.000 CDU/ml Collagenase I (Sigma, Steinheim, Germany), 32 mg/ml Dispase II (Roche Diagnostic, Mannheim, Germany) and 5 MU/ml DNase I (Calbiochem, Darmstadt, Germany) for 40 min at 37°C and then passed through a 70-µm nylon mesh filter (BD Biosciences, Erembodegem, Belgium). Cells were incubated at 4°C for 40 min in PBS with 2% FCS, in the presence of appropriate dilutions of labeled monoclonal antibodies: anti-mouse MHCII-PE (Clone M5, eBioscience, Frankfurt, Germany), anti-CD19-PE-Cy5.5 (Clone 6D5, Beckman Coulter, Krefeld, Germany), anti-F4/80-APC (Clone BM8, eBioscience), and anti-Ly6G-PE (Clone 1A8, BD Biosciences). Stained cells were subsequently analyzed, using an Accuri C6 Cytometer and FACS analysis software CFlow Version 1.0.227.4 (Accuri Cytometers, Inc. Ann Arbor, MI USA).

## Results

### Viral replication and viability of the human hepatocellular carcinoma cells HuH7 and PLC after infection with GLV-1h68 in culture

**Figure 1:**
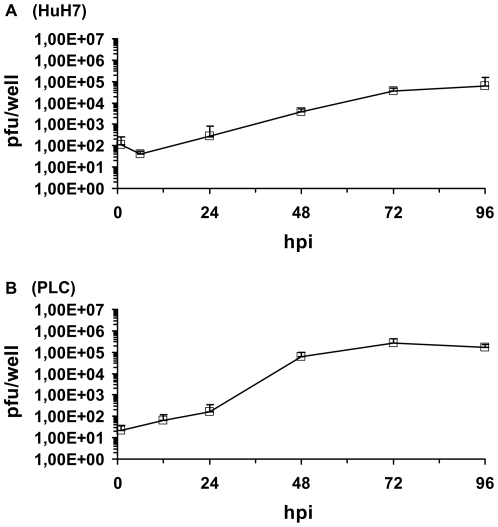
Replication efficiency of vaccinia virus strain GLV-1h68 in HuH7 (A) and PLC (B) cells at an MOI of 0.1. Supernatants were collected from virus-infected cells at various time points (hours) post-infection (hpi). Viral titers were determined as pfu per well in triplicates. Averages plus standard deviation are plotted.

The replication efficiency of GLV-1h68 in HuH7 and PLC cells was analyzed as described in Materials and Methods. As a result, GLV-1h68 was found to replicate efficiently in both hepatocellular carcinoma cell lines HuH7 (Fig. 1A) and PLC (Fig. 1B). The highest virus titer was identified in wells of virus-infected PLC cells at 72 h post infection (2.7×10^5^ pfu/well).

**Figure 2:**
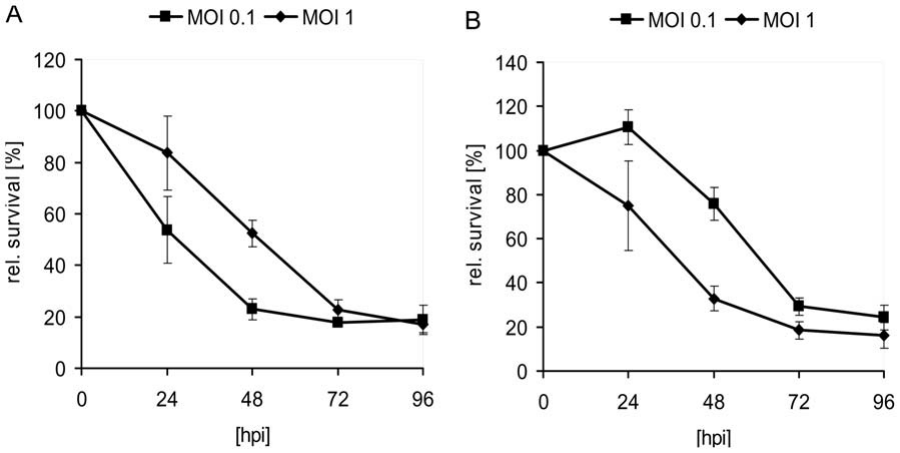
Viability of hepatocellular carcinoma HuH7 (A) and PLC (B) cells after GLV-1h68 infection at MOIs of 0.1 and 1.0, respectively. Viable cells after infection with GLV-1h68 were determined by use of 3-(4,5-dimethylthiazol-2-yl)-2,5-diphenyltetrazolium bromide (MTT) (Sigma, Taufkirchen, Germany). Mean values (n = 3) and standard deviations are shown as percentages of respective controls.

In order to test the ability of the GLV-1h68 virus to infect and lyse HuH7 and PLC cells we performed a cell viability assay (Fig. 2). Ninety-six hours after GLV-1h68 infection at MOIs of 0.1 and 1.0, 18.7% and 17.2% of the HuH7 cells (Fig. 2A) and 24.2% and 16.2% of the PLC cells (Fig. 2B) survived the treatment, respectively.

To confirm the efficient infection and replication of GLV-1h68 in hepatocellular carcinoma HuH7 and PLC cells, we followed the virus-mediated expression of the *Renilla* luciferase-green fluorescent protein (Ruc-GFP) fusion protein by fluorescence microscopy. In this experimental setting we found that infection with GLV-1h68 at an MOI of 1.0 exhibited the strongest GFP expression at 96 h in both HuH7 and PLC cells (Figure S1). At this time point most dead/dying cells (detectable by positive propidium iodide (PI) staining) were observed.

These results indicated that GLV-1h68 was able to efficiently infect and kill both, HuH7 and PLC cells in cell culture.

### Effects of the GLV-1h68 treatment *in vivo*

**Figure 3:**
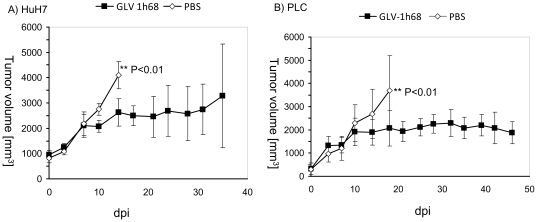
Growth of HuH7 and PLC tumors in GLV-1h68- and mock-treated mice. Groups of HuH7 (Fig. 5A) or PLC tumor-bearing nude mice (Fig. 5B) were either treated with a single dose of 5×10^6^ pfu GLV-1h68 (n = 5) or with PBS (mock control, n = 5). Tumor size was measured twice a week. Two-way analysis of variance (2way ANOVA) with Bonferroni post-test was used for comparison of two corresponding data points between groups. P<0.05 was considered statistically significant. ** P<0.01. In addition, statistical power was calculated post-hoc using G*power 3.1.2 software (http://www.psycho.uni-duesseldorf.de/abteilungen/aap/gpower3/). The statistical power for alpha  = 0.05 was 75%, for alpha = 0.01 42%.

To test the therapeutic efficacy of GLV-1h68 against human hepatocellular carcinoma *in vivo*, nude mice at the age of 6-8 weeks were implanted with either HuH7 or PLC tumor cells. Ten days after transplantation, tumor-bearing mice were intravenously injected either with 5×10^6^ pfu of GLV-1h68 (n = 5) or with PBS only (n = 5) and were monitored for tumor growth twice a week (Fig. 3). As a result, intravenous injection of GLV-1h68 was found to significantly inhibit the growth of both HuH7 (Fig. 3A) and PLC tumors (Fig. 3B) at 21 dpi. Since one mouse of the virus-injected HuH7 xenograft group developed a tumor volume greater than 3000 mm^3^ at 35 dpi we prematurely had to terminate the study with this verum group. In contrast, all virus-treated PLC xenografted mice showed a significant tumor reduction compared to controls by the end of the study (at 46 dpi) (Fig. 3B).

### Analysis of tumor phenotype differences in GLV-1h68-infected and uninfected HuH7/PLC xenograft-bearing animals by visual inspection, immunohistochemistry and FACS

**Figure 4:**
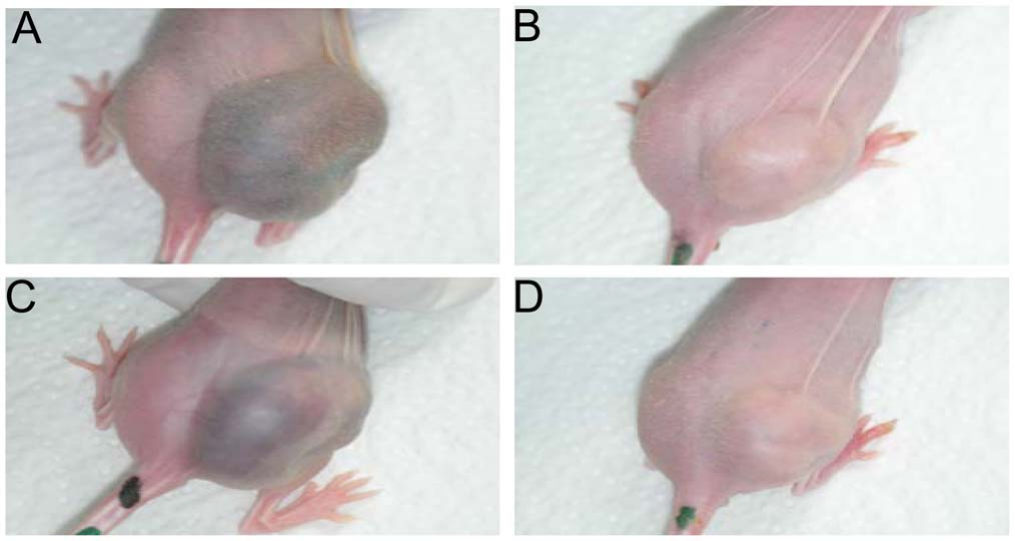
Phenotypic difference between GLV-1h68- and non-treated PLC-tumor-bearing mice. Pictures were taken at day 18 after injection with PBS (mock) (A, C) or GLV-1h68 (B, D).

**Figure 5:**
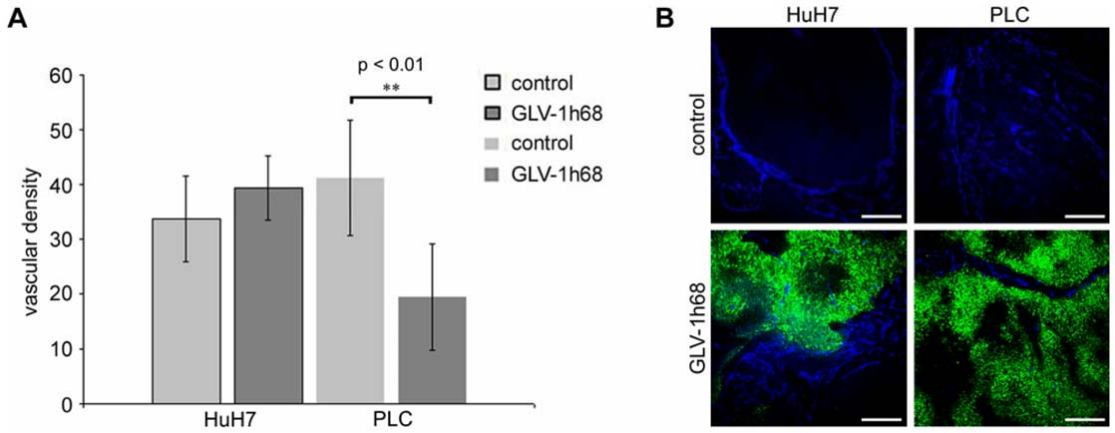
Analysis of GLV-1h68 virus-induced changes in HuH7- or PLC-tumor vascularization by confocal laser microscopy. **Determination of vascular density using CD31 immunohistochemistry in virus- treated and non-treated HuH7 or PLC, tumors (A).** The vascular density was measured in CD31-labeled tumor cross-sections (n = 6 per group) and presented as mean values +/− standard deviations. The asterisks (**) indicate a significant difference between experimental groups (** P<0.01; Student's *t*-test). **Confocal images of virus-treated and non-treated HuH7- or PLC-tumors (B).** Tumor vasculature was labeled with anti-CD31 antibody (blue) and viral infection was indicated by GFP fluorescence (green). Scale bars represent 300 µm.

**Figure 6:**
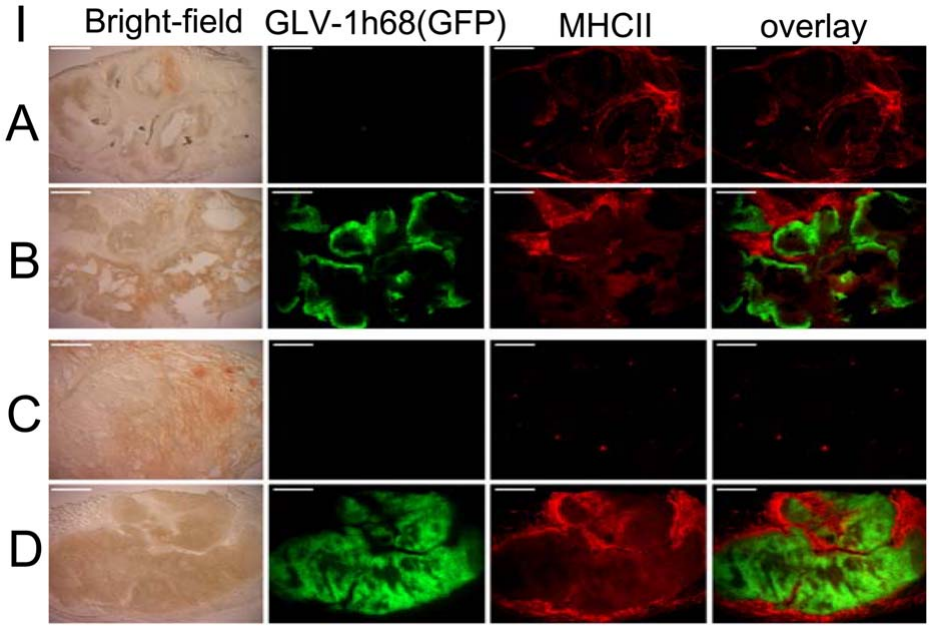
Immunohistochemical staining of MHC class II-positive cells in GLV-1h68-infected and uninfected HuH7 or PLC xenograft tumors at 10 dpi. Mice bearing tumors of HuH7 (A, B) or PLC (C, D) origin either were mock treated (A, C) or infected with GLV-1h68 (B, D). Tumor sections were labeled with an anti-MHCII antibody (red) and viral infection was indicated by GFP fluorescence (green). In addition, overlays of MHCII and GFP signals and transmission images (bright-field) are shown. Scale bars represent 1 mm.

In the animal studies previously described, we observed a phenotypic difference (switch) in the tumor appearance of virus-infected versus mock-treated PLC tumor-bearing mice by day 14 p.i. The control mice developed dark bluish, hemorrhagic tumors as shown in (Fig. 4A, C), the PLC tumors with GLV-1h68, however, remained small and exhibited light colors (Fig. 4B, D). In contrast to PLC tumors, no hemorrhagic phenotype was observed in the uninfected HuH7 tumors (data not shown). Histological and immunological analysis was made to study the phenotypic differences in two groups (n = 4 per group) of virus-infected and uninfected PLC mice. Tumorous mice were sacrificed at 10 dpi and using CD31 immunohistochemistry tumor vasculature in GLV-1h68-treated and control-PLC tumors was quantified (Fig. 5). The data showed a significant (**P<0.01) decrease in the number of blood vessels in virus-treated PLC xenografts in comparison to controls at day 10 after virus injection (Fig. 5A). HuH7 tumors treated with GLV-1h68, however, exhibited no difference in density of the tumor vasculature. The drastic change in tumor vascularization in virus-treated PLC tumors may explain the loss of the hemorrhagic phenotype. Subsequently, we also analyzed the cell entry of GLV-1h68 virus into and the presence of host immune cells in tumor tissue of virus-infected and uninfected mice (Fig. 6). Ten-days post-infection HuH7 and PLC tumors both revealed an intense intratumoral virus colonization and a specific peri- and intra-tumoral infiltration of MHC class II-expressing host cells (monocytes/macrophages, dendritic cells and B cells) surrounding virus-infected cancer cells (Fig. 6). Interestingly, we found more extensive viral load in the tumor tissues of PLC tumor-bearing mice (Fig. 6D, GFP) compared to the HuH7 tumor-bearing mice at 10 dpi (Fig. 6B, GFP), as indicated by the amount of GFP detection. In addition, MHC class II-positive host cells were absent in the uninfected PLC tumors (Fig. 6C, row 3). In contrast, a strong peri-tumoral recruitment of MHC class II-positive cells was observed in uninfected HuH7 tumors (Fig. 6A, row 3).

These immunohistological data were also quantitatively analyzed and verified by flow cytometric analysis (FACS) of tumor single cell suspensions derived from infected and uninfected HuH7 and PLC tumors at 7dpi (Table 1). The amount of MHCII^+^, F4/80^+^, CD19^+^ and Ly6G^+^ cells were much higher in cells derived from virus-infected tumors than from uninfected controls. The increased accumulation of host immune cells after virus injection was observed in both xenograft models (Table 1). Interestingly, the presence of F4/80^+^, MHCII^+^, CD19^+^ and Ly6G^+^ was 2.79- to 12.98-fold higher in uninfected HuH7 mice compared to uninfected PLC mice (Table 1). We found also 5.91-fold more GFP-positive cells in infected PLC tumors upon comparison to infected HuH7 tumor at 7dpi (Table 1).

**Table 1:** FACS characterization and comparison of tumor single cell suspensions derived from infected and uninfected HuH7 and PLC tumors.

Tumor^B^	GLV-1h68/untreated Ratio (HuH7)	GLV-1h68/untreated Ratio (PLC)	uninfected HuH7/uninfected PLC
**Marker** A			
MHCII	2.28	5.68	4.68
F4/80	1.01	3.34	2.79
CD19	1.56	5.63	6.41
Ly6G	1.86	10.22	12.98
CTotal virus-infected cells (GFP-positive in %)	1.20% +/− 0.012%	7.10% +/−0.044%	

A Markers: MHCII-PE antibody detects a polymorphic determinant present on B cells, monocytes, macrophages and dendritic cells.

F4/80-APC antibody recognises the F4/80 antigen, that is expressed by a majority of mature macrophages and is the best marker for this population of cells.

CD19 is expressed on B cells and follicular dendritic cells.

Ly6G-PE, also known as Gr-1- antigen, is expressed on mouse neutrophils, predominantly granulocytes.

B Single cell suspensions derived from infected and uninfected HuH7 and PLC tumors 7dpi (n = 4 for uninfected and n = 3 for virus-infected groups) were used for FACS characterization. Ratios greater than 1 indicate an increased accumulation of host immune cells.

C GFP-positive cells of the infected tumors were presented as mean values (n = 3) +/− standard deviations in percentages.

The increased presence of host immune cells in tumor tissue before virus injection might be responsible for the different infection and therapeutic efficacies of GLV-1h68 in these two xenograft models.

### Mouse immune-related protein profiling of infected and uninfected HuH7 and PLC derived tumors

In order to study the influence of the tumor microenvironment on the efficiency of cancer therapy, we analyzed and compared the protein profiles of infected and uninfected HuH7 and PLC tumors. Lysates of tumors were prepared and aliquots used for examination of the expression levels of immune-related proteins of mouse origin, as described in Materials and Methods. Data showed that, GLV-1h68 injection led to an increased production of most of the pro-inflammatory cytokines and chemokines tested (Table 2). Many of these cytokines and chemokines, such as IP-10, IL-6, IL-12, IL-18, MCP-1, MCP-3, MCP-5, M-CSF, TNF-alpha etc., are known to activate macrophages, monocytes, neutrophils, eosinophils, etc., and to trigger pro-inflammatory responses in target tissues. In contrast, only MIP-1-gamma was highly down-regulated upon virus injection in both xenograft models. In addition, our protein profile data revealed a 6.4- to 22.2-fold down-regulation of coagulation factors such as fibrinogen and factor VII, concerning the ratio of GLV-1h68 treated/untreated PLC human hepatoma xenografts when compared with the ratio of GLV-1h68 treated/ untreated HuH7 human hepatoma xenografts at 10 dpi (Table 2).

**Table 2:** Comparison of mouse immune-related protein antigen profiling in primary PLC and HuH7 tumors with or without GLV-1h68 at day 10 after virus injection (n = 3).

Antigen	GLV-1h68 / untreated Ratio (HuH7)	GLV-1h68 / untreated Ratio (PLC)	Classification
GM-CSF	2.18	6.23	granulocyte-macrophage colony-stimulating factor
IFN-gamma	1.29	1.24	proinflammatory cytokine
IL-6	5.67	11	proinflammatory cytokine
IL-12 (IL-12p70)	3	4.27	pleiotropic cytokine
IL-18	1.2	3.7	proinflammatory cytokine
IP-10 (CXCL10)	3.12	112.2	interferon-gamma-induced protein
MCP-1 (CCL2)	6.55	40.9	proinflammatory cytokine
MCP-3 (CCL7)	2.06	26.6	proinflammatory cytokine
MCP-5 (CCL12)	18.17	40.6	proinflammatory cytokine
MPO	0.9	70	myeloperoxidase
M-CSF-1	1.15	5.23	proinflammatory cytokine
MIP-1beta	1.59	6.57	proinflammatory cytokine
MIP-2 (CXCL2)	0.81	15.44	proinflammatory chemokine
TNF-alpha	1.2	2.91	proinflammatory cytokine
MIP-1gamma (CCL9)	0.03	0.0227	macrophage inflammatory protein
Factor VII	1.045	0.047	plays a role in coagulation cascade
Fibrinogen	0.812	0.126	plays a role in coagulation cascade
GST-alpha	0.92	0.0071	biomarker
Haptoglobin	1.632	0.17	biomarker

All ratios greater than 1 indicate an up-regulation of the protein expression, and all ratios less than 1 indicate down-regulation.

Thus, the drastic reduction of fibrinogen and factor VII in the course of application of GLV-1h68 could also be an explanation for the loss of the hemorrhagic phenotype in virus-treated PLC human hepatoma xenografts.

Lastly, we also compared the protein profiles of uninfected HuH7 and PLC derived tumors (Table 3). Interestingly, several significant differences in the expression levels of factors such as CD40, factor VII, GST-alpha, haptoglobin and MPO were observed (Table 3).

**Table 3:** Comparison of mouse immune-related protein antigen profiling in uninfected primary HuH7 and PLC tumors at day 20 after implantation (n = 3).

Antigen (least detectable dose *)	HuH7 tumor tissue	PLC tumor tissue	Classification
CD40 * 3.3 pg/ml	272 pg/ml	109 pg/ml	a type I glycoprotein belonging to the TNF receptor superfamilyjavascript:void(0);
Factor VII (FVII) * 3.5 ng/ml	198 ng/ml	851 ng/ml	plays a role in coagulation cascade (increased FVII indicates blood vessel injury)
Fibrinogen * 0.89 µg/ml	38 µg/ml	300 µg/ml	plays a role in coagulation cascade
GST-alpha * 0.083 ng/ml	6.3 ng/ml	851 ng/ml	biomarker of hepatocyte injury
Haptoglobin * 0.072 µg/ml	0.98 µg/ml	14 µg/ml	biomarker
MPO * 0.21 ng/ml	822 ng/ml	25 ng/ml	myeloperoxidase

*The least detectable dose was determined as the mean + 3 standard deviations of 20 blank readings.

## Discussion

Approximately 7% of all newly diagnosed cancers worldwide are liver cancers with the third most common cause of death worldwide (http://globocan.iarc.fr/factsheets/cancers/liver.asp). The increasing incidence and poor prognosis of hepatocellular carcinoma [1], [17] emphasizes the urgent need to find novel therapies for HCC (http://globocan.iarc.fr).

In this study, we investigated the oncolytic efficiency of the vaccinia virus strain GLV-1h68 against the two hepatocellular carcinoma cell lines HuH7 and PLC in culture and the therapeutic efficacy in xenograft models. The results showed that GLV-1h68 was able to effectively infect, replicate in, and lyse hepatocellular carcinoma cells in culture. The efficiency of viral replication correlated well with degree of cell lysis and with expression of the marker *Renilla* luciferase-green fluorescent protein fusion protein. In addition, the current study also demonstrated the suitability of GLV-1h68 to achieve a highly effective form of virotherapy in mice. We also observed a significant inhibition of tumor growth and damage to the tumor tissues in the GLV-1h68-treated HuH7 and PLC tumor-bearing mice when compared to untreated controls.

More importantly, the data clearly demonstrated that the optimal oncolytic effect of the GLV-1h68 is dependent on the interactions with the components of the tumor microenvironment, such as tumor vasculature and with the cells of the host immune system. The finding of the phenotypic switch in virus-infected PLC-xenograft tumors revealed a significant decrease in the number of blood vessels when compared to controls at day 10 after virus injection (P<0.01). The vascular density in infected HuH7 tumors, however, did not change in comparison to uninfected controls, which were non-hemorrhagic.

The protein profiling data of infected and uninfected PLC tumors (Table 2) clearly revealed that this process was associated with a strong induction of cytokines like TNF-α, IL-12 and IP-10 that are known to have negative effects on vascularization [18], [19], [20]. We also found a significant down-regulation of factor VII, fibrinogen, glutathione S-transferase alpha (GST-alpha), and haptoglobin in GLV-1h68-colonized PLC tumors only (Table 2). Increased levels of these proteins are found upon injury to blood vessels or interference with hemorrhagic process (Table 3, classification). Taken together, these data did provide direct evidence for the anti-vascular and anti-hemorrhagic effects of GLV-1h68-virus, which may be responsible for the phenotypic switch of PLC-tumor xenografts. In contrast, no hemorrhagic tumor phenotype was observed in HuH7 xenografts. Molecular mechanisms causing such differences between PLC and HuH7 tumors in nude mice are currently unknown.

Injection of GLV-1h68 to tumorous mice led to inhibition of tumor growth in both HuH7 and PLC xenografts at 21 dpi and beyond. Therefore, we investigated which components of the tumor microenvironment may play a crucial role in the optimal oncolytic effect of GLV-1h68-virus. In this context, we analyzed the virus colonization and the presence of host immune cells in the tumor tissues of virus-infected and uninfected HuH7- and PLC-tumor bearing mice at 7 and 10 dpi (Fig. 6, Table 1). We found a more extensive viral load in the tumor tissues of PLC mice in comparison to the HuH7 mice at 7 and 10 dpi (Fig. 6, Table 1). Further, at these time points we also observed a 2.79- to 12.98-fold higher peri-tumoral infiltration of MHC class II-expressing host cells especially granulocytes and CD19+ in uninfected HuH7 mice than in uninfected PLC mice (Fig.6, Table 1). These findings were also confirmed by mouse protein profiling experiments. Approximately a 2.5- to 33-fold higher presence of CD40- or MPO-positive cells were located in uninfected HuH7 tumors upon comparison with uninfected PLC tumor (Table 3). The marker CD40 is constitutively expressed by antigen presenting cells, including dendritic cells, B cells and macrophages [21]. MPO is an enzyme, most abundantly present in neutrophil granulocytes [22]. The increase in MHC class II-expressing immune cells which surround HuH7 tumor tissues already before virus treatment may be responsible for the lower tumor colonization shortly after virus injection resulting possibly in less efficient therapeutic effect of GLV-1h68 in the HuH7 mice, but not in PLC xenografts. The differences between PLC and HuH7 tumors might be due to the different expression levels of cyclooxygenase-2 (COX-2) in these cancer cell lines [23]. It has been shown that COX-2 is associated with carcinogenesis in hepatocellular carcinoma [24], [25] and an elevated COX-2 levels led to inflammation in liver tissues [26]. Such high COX-2 expression in HuH7 tumors might be responsible for stronger peri-tumoral infiltration of MHC class II-expressing host cells in comparison to PLC tumors. The observed heterogeneity of tumor tissues indicates the need for analysis of the tumor microenvironment which may help to design optimized therapeutic strategies. In our opinion an optimally designed vaccinia virus therapy needs to consider the interplay among the components of the tumor microenvironment.

In summary, use of GLV-1h68 strain demonstrated outstanding anti-tumor and anti-vascular effects in PLC and lesser efficacy in HuH7 hepatocellular carcinoma xenografts. Therefore we propose that GLV-1h68 strain is a very potent live drug in preclinical studies to be used soon for the treatment of primary liver cancer in humans. Moreover, results of a Phase 1 study of intravenous administration of GL-ONC1 (GLV-1h68) Vaccinia virus in patients with advanced solid cancer demonstrated acceptable safety, preliminary evidence of anticancer activity and virus replication in several patients (positive for GL-ONC1 viral plaque assay and GFP imaging; (http://www.ncri.org.uk/ncriconference/2010abstracts/abstracts/C122.htm).

## Supporting Information

Figure S1 Figure S1 shows the effects of GLV-1h68 virus infection on HuH7 and PLC cells. Hepatocellular carcinoma cells HuH7 (Fig. 1A) and PLC (Fig. 1B) were infected with GLV-1h68 at an MOI of 1.0 followed by monitoring of virus-mediated expression of the Ruc-GFP fusion protein by fluorescence microscopy. (**BF**) Transmitted light view of virus-infected cells; (**GFP**) Expression of GFP in infected cells detected by direct fluorescence; (**PI**) Propidium iodide staining of dead cells; (**Hoechst)** Nuclear staining; (**Merged**) Co-localization of GFP with the dead cells. All pictures in this set were taken at the same magnification. Scale bars represent 0.25 mm.
(TIF)
