# Supplementary figures and images for: Efficient Colonization and Therapy of Human Hepatocellular Carcinoma (HCC) Using the Oncolytic Vaccinia Virus Strain GLV-1h68
Source: PLoS One. 2011 Jul 11;6(7):e22069. doi: 10.1371/journal.pone.0022069 (PMC3133637; doi:10.1371/journal.pone.0022069)

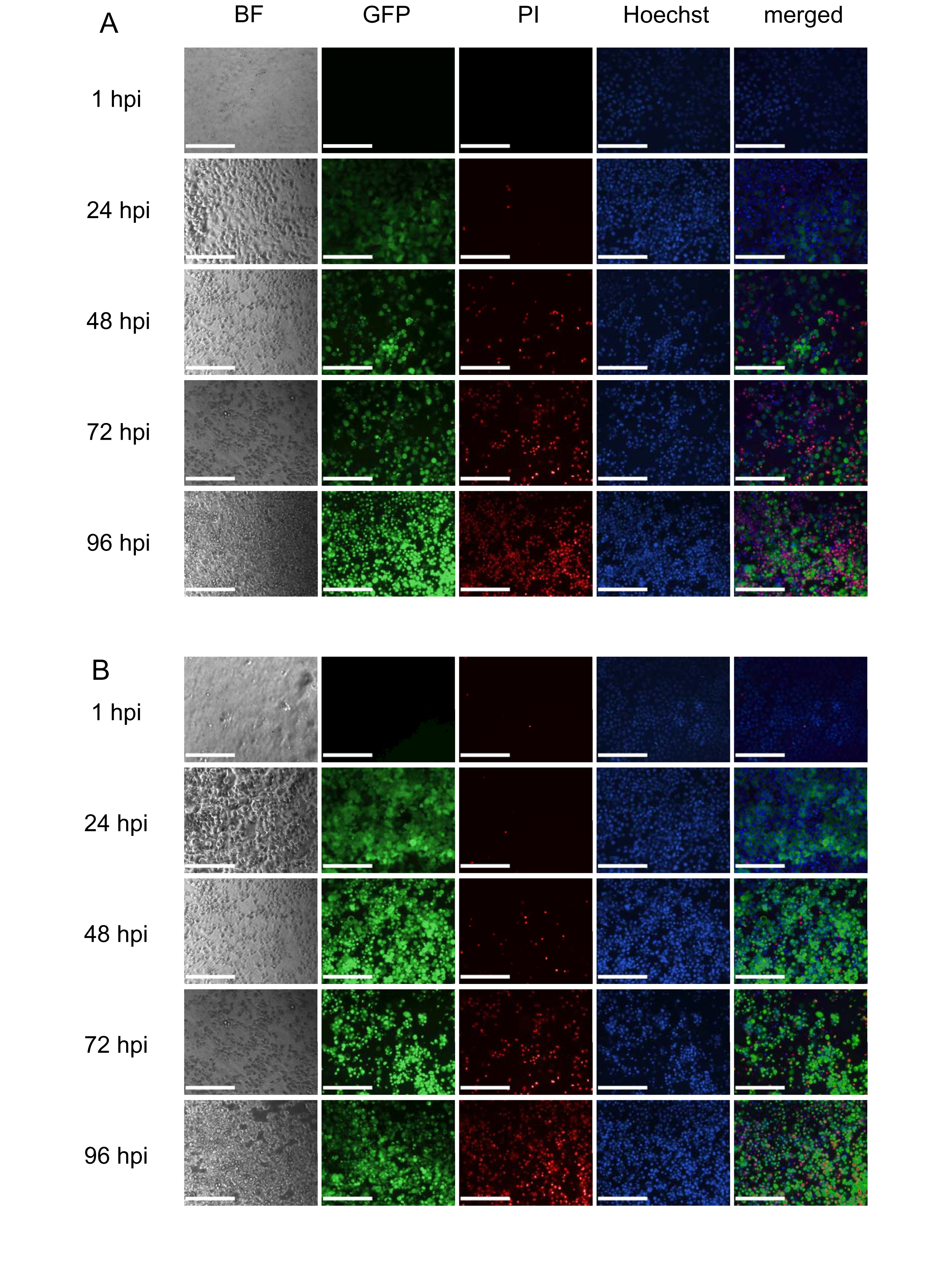

Supplement: Figure S1 — shows the effects of GLV-1h68 virus infection on HuH7 and PLC cells. Hepatocellular carcinoma cells HuH7 (Fig. 1A) and PLC (Fig. 1B) were infected with GLV-1h68 at an MOI of 1.0 followed by monitoring of virus-mediated expression of the Ruc-GFP fusion protein by fluorescence microscopy. (BF) Transmitted light view of virus-infected cells; (GFP) Expression of GFP in infected cells detected by direct fluorescence; (PI) Propidium iodide staining of dead cells; (Hoechst) Nuclear staining; (Merged) Co-localization of GFP with the dead cells. All pictures in this set were taken at the same magnification. Scale bars represent 0.25 mm. (TIF) [file pone.0022069.s001.tif]
